# Development and evaluation of short-form version of the Constitution in Chinese Medicine Questionnaire: study a new and best brief instrument of Chinese medicine for health management

**DOI:** 10.1186/s13020-023-00844-3

**Published:** 2023-10-30

**Authors:** Ming-Hua Bai, Zhu-Qing Li, Huai-Yu Wang, Xiao-Li Ma, Zhong-Li Wang, Shi-Jun Li, Si-Ying Dong, Zi-Ling Zhang, Wen-Le Li, Shun-Qi Chen, Yu-Yang Cai, Xiao-Shan Zhao, Ji Wang, Qi Wang

**Affiliations:** 1 National Institute of TCM, Constitution and Preventive Medicine, Beijing University of Chinese Medicine, Beijing, 100029 China; 2 College of Nursing, Hebei University, Baoding, 071000 China; 3 College of Nursing, Jiujiang University, Jiujiang, 332000 China; 4 Pharmacy Department, The NO.5 Hospital of Baoding, Baoding, 071000 China; 5 School of Chinese Medicine, Southern Medical University, Guangzhou, 510515 China

**Keywords:** Constitution in Chinese Medicine Questionnaire, Body constitution, Short-form, Psychometric property evaluation, Diagnostic validity

## Abstract

**Background:**

More efficient instruments for body constitution identification are needed for clinical practice. We aimed to develop the short-form version of the Constitution in Chinese Medicine Questionnaire (CCMQ) and evaluate for health management.

**Methods:**

First, the short forms were developed through expert survey, classical test theory (CTT), and modern item response (IRT) based on the CCMQ. A combination of e-mail and manual methods was used in expert survey. Then, five indexes of CTT including criteria value-critical ratio, correlation coefficient, discrete tendency, internal consistency, and factor loading were used. And, IRT method was used through analyzing the discrimination and difficulty parameters of items. Second, the three top-ranked items of each constitution scale were selected for the simplified CCMQ, based on the three combined methods of different conditions and weights. Third, The psychometric properties such as completion time, validity (Construct, criterion, and divergent validity), and reliability (test–retest and internal consistency reliability) were evaluated. Finally, the diagnostic validity of the best short-form used receiver operating characteristic (ROC) curve.

**Results:**

Three short-form editions were developed, and retained items 27, 23 and 27, which are named as WangQi nine body constitution questionnaire of Traditional Chinese Medicine (short-form) (SF-WQ9CCMQ)- A, B, and C, respectively. SF-WQ9CCMQ- A is showed the best psychometric property on Construct validity, Criterion validity, test–retest reliability and internal consistency reliability. The diagnostic validity indicated that the area under the ROC curve was 0.928 (95%CI: 0.924–0.932) for the Gentleness constitution scale, and were 0.895–0.969 and 0.911–0.981 for unbalance constitution scales using the cut-off value of the original CCMQ as 40 (“yes” standard) and 30 (“tendency” standard), respectively.

**Conclusions:**

Our study successfully developed a well short-form which has good psychometric property, and excellent diagnostic validity consistent with the original. New and simplified instrument and opportunity are provided for body constitution identification, health management and primary care implementation.

## Background

In the 1970s, a new branch of Traditional Chinese Medicine (TCM), the Constitutional Medicine of TCM, was developed by Pro.Wang Qi; this branch expresses the principle behind Chinese health medicine and individualized treatment and provides corresponding methods [1]. The constitutional theory of TCM establishes a new concept of health, which indicates that health refers to the good adaptation of an individual’s physical and psychological being to the natural and social environment during the whole life span [2]. It reflects an individual’s current and future health trends in four aspects including physical differences, life processes, psychological condition, and adaptability to natural and social environments [3]. With the progress of the research on the association between body constitution (BC) and diseases, it provides evidence supporting the BC identification to carry out disease prevention and treatment and promote population health in TCM clinical and public health practice [4]. Health management based on BC identification of TCM has been incorporated into China’s Basic Public Health Services system and used nationwide for primary care. Moreover, in other countries, such as Japan [5], South Korea [6, 7], Thailand [8, 9], Singapore [10, 11], Malaysia [12] in Asia, as well as in Europe [13, 14], America [15, 16], Africa [17], and Oceania [18], it has been promoted and applied [19].

As a basic and paramount instrument of BC identification and determination, *the Constitution in Chinese Medicine Questionnaire* (CCMQ) was developed by Prof. WangQi’s group based on the constitutional theory of TCM [20] and became the standard of the *China Association of Chinese Medicine* (CACM) in 2009 [21]. CCMQ has 60 items measuring 9 BC types: Gentleness constitution (GTC), *Qi*-deficiency constitution (QDC), *Yang*-deficiency constitution (YaDC), *Yin*-deficiency constitution (YiDC), phlegm-dampness constitution (PDC), damp-heat constitution (DHC), blood-stasis constitution (BSC), *Qi*-stagnation constitution (QSC) and inherited-special constitution (ISC) [21]. Psychometric properties of CCMQ have been confirmed [22, 23] and applied in public health management nationwide, and proved to be fruitful in China [3]. In addition, other editions such as the Hong Kong edition [24], English edition [15, 25, 26], Korean edition [27], Japanese edition [28] and so on are popularized worldwide [13].

More items of long-form questionnaire can identify and determine better. However, with the development of society and the transformation of the medical model, disadvantages of the long-form questionnaire appeared, such as time-consuming, loss of patience and high incomplete rate. Thus, it is necessary to develop a short version of the questionnaire [29]. For example, SF12, SF8, and SF6D were developed based on SF36 [30], and WHOQOL-BEEF was developed based on WHOQOL [31–33]; these versions are all simplified on the basis of the long scale. CCMQ also has the same problems and deficiencies [34, 35], so the development a simplified version has been concerned [34] and attempted [36–39].

In the completed attempts to simplify CCMQ, methods, such as classical test theory (CTT) alone [37–39] and CTT combined with modern item response theory (IRT) [40], have been tested, and the importance of expert opinions has also been noted [36]. Several simplified versions of the development have been attempted, and have achieved certain success; psychometric properties are acceptable, thereby improving the efficiency of filling and application promotion. However, some shortcomings and unsatisfactory exist, such as some items are difficult to improve in BC intervention so it cannot reflect the intervention effect well, the overall representatives for the Chinese population are insufficient, and the attention to experts is not enough [36, 37, 39, 40], *Classification and Determination of Constitution in TCM* (CDCTCM) was used instead of the formal CCMQ for data collection and short form development [38], etc.

To address the above deficiencies, this study developed a simplified version based on the verification and evaluation of the large sample data of CCMQ, combined with CTT and IRT, and give more attention to the experience of experts than before through considering expert opinions at the same time as other methods. It is expected to provide a simpler and more effective assessment instrument for the identification of BC of TCM.

## Methods

### Development on short-form versions

#### Study group

A study group was formed at first, which consists of a core group, an expert group, and an investigation group. Three groups were mainly responsible for the idea and design of the study, academic support and consultation, and distribution and recycling of questionnaires.

#### Face-to-face expert consultation

A face-to-face expert discussion meeting was held for consultation. Experts for the conference were from the Constitutionology of TCM, Diagnostics of TCM, Psychometrics, Methodology, and Medical Statistics, etc. In the conference, the design for simplifying CCMQ was argued, and the integrated approach to simplify scales constituting of expert survey method, CTT, and IRT has been recognized by experts.

#### Expert survey

##### Survey questionnaire for expert

The *Expert Questionnaire of CCMQ* (EQ-CCMQ) was compiled based on CCMQ. The EQ-CCMQ mainly includes the following contents. a) Preface, mainly explain the research and expert questionnaire, b) Basic information, record the basic information of participating experts, c) Application information, collect intention information, d) Expert opinions and suggestions, select and rank the top 4 most important items for each BC type scale (only the top 3 most important items for each scale would be retained), and e) All other comment and recommendation information from experts.

##### Implementation of survey

During the implementation of the survey study, a combination of e-mail and manual methods was used. Purposive sampling was conducted. Experts surveyed by e-mail are authors of academic or research articles published about the Constitutionology of TCM in the last 3 years, representing the most active experts in this subject. Additionally, experts surveyed by manual-questionnaire are a team of experts in original CCMQ development. The experts were categorized into the manual questionnaire survey group if who can be classified into both two groups.

##### Scoring method

According to the method determined by the expert group and face-to-face expert consultation, the data of the expert survey were scored and weighted. (a) Points are scored for the number of times an item is selected into each of the top 3 by experts, and one point is counted for each time it is selected, respectively. (b) The point of the item is weighted, and the first important item is given 3* points value, the second important item is given 2* points value, and the third important item is given 1 * points value [41]. (c) The weighted scores obtained of each item in step b are added, and the sum is the weighted total score. (d) The 3 items with the highest total score will be retained for the simplified questionnaire. In addition, the item that needs to be added in the experts’ opinion, if it is recommended once, will be recorded as 1 point, and the item will be marked as item X in the relevant constitution type.

#### Classical test theory (CTT)

##### Data of sample

All data were obtained from the system of one technology institution and collected from August 2015 to October 2017. All data were anonymized to protect the privacy. The study sample consisted of 94,718 cases aged from 15 to 64 years old and had BC types classified and determined by CCMQ [20, 42] and CDCTCM [21] promulgated by CACM. There were 38,132 males (40.26%) and 56,586 females (59.74%).There were 52,632 patients with specific age in the study, the average age was 39.88 ± 10.258 (15–64 years old). A total of 35,783 cases were filled in with provincial data. The research group obtained samples from North China (Beijing, Tianjin, Shanxi, Hebei and Meimenggu) accounted for 9.39% (3359 case), East China (Shanghai, Jiangsu, Zhejiang, Anhui, Jiangxi, Shandong, Fujian and Taiwan) accounted for 24.36% (8715), Central China (He’nan, Hubei and Hu’nan) accounted for 10.25% (3666), South China(Guangdong, Guangxi, Hainan, HongKong and Macao) accounted for 5.99% (2145), Southwest China(Chongqing, Sichuan, Guizhou, Yunnan and Xizang) accounted for 24.39% (8728), Northwest China(Shanxi, Gansu, Qinghai, Ningxia and Xinjiang) accounted for 24.18% (8653), and Northeast China (Heilongjiang, Jilin and Liaoning) accounted for 1.44% (517).

##### Methods for simplification

Five methods of CTT including criteria value-critical ratio (CVCR), correlation coefficient, discrete tendency, internal consistency, and factor loading were used comprehensively. (a) CVCR method. Independent sample t test was used to screen items. Statistical analysis was conducted on the differences between the high and low groups in the BC score, which each accounted for 27% of the sample size. The item will be deleted when *P* > 0.05 and t < 3.000, then the top three discrimination ability items would be reserved [43]. (b) Correlation coefficient method. Correlation analysis was carried out between item-scale of each BC type, and items with a correlation coefficient < 0.4 will be discarded for low representativeness.[44]. (c) Discrete tendency method. The mean and standard deviation (SD) of each item score was calculated. The item will be considered for deletion when SD < 1.00 [45, 46]. (d) Internal consistency method. Cronbach’s alpha coefficient of each BC type was calculated. When the Cronbach’s alpha coefficient increases significantly after the deletion of the item, this item will not be considered for retention in the new questionnaire [36, 47, 48]. (e) Factor loading. The factor loading of the item in the affiliated BC type scale was used as the basis for item selection. It will be deleted when the factor loading coefficient of the item is < 0.4 [43].

### Item response theory (IRT)

As a widely used method for the scale development, IRT pays more attention to the performance of subjects in specific items, which compensates for the deficiencies of CTT. IRT is used to develop a short-form version by analyzing the discrimination and difficulty parameters of CCMQ. (a) Unidimensionality test. Principal component analysis in exploratory factor analysis (EFA) was used [49]. If the eigenvalue of the first factor is more than 3 times that of the second factor in EFA, it can be considered that the scale content conforms to the unidimensional assumption and IRT analysis can be used [50]; (b) discrimination parameters a. If a < 0.5, the item discrimination is not well and can be deleted [51]; (c) difficulty parameters b. If b > 3, the item difficulty is relatively high and can be deleted [52]; and (d) considering the discrimination and difficulty of the reserved items comprehensively, the 3 items with the best parameters a and b were selected as the alternative items of the short-form CCMQ.

#### Selecting methods

The short-form version of CCMQ was developed by 3 methods, which consist of the ES method, CTT and IRT. A total of 8 indices had been selected, including (a) the expert score in the expert survey; (b) the CVCR, correlation coefficient, discrete tendency, internal consistency coefficient, and factor loading in CTT, as well as; and (c) differentiation and difficulty parameters in IRT. The above indicators were used to statistical analysis and item selection. According to the method in literature [36, 53] and expert opinions in the design stage, 3 short forms of CCMQ were developed by selecting and retaining the top 3 important items of each of BC type scale based on the combination of different conditions and weights. (Table 1).

**Table 1:** Steps of selecting methods

Selecting methods	Steps
Method 1	Step1: The 8 indexes were selected as independent variables, and the top 3 items were selected Step2: The items with the highest frequency in the top 3 of each index are selected to form the simply questionnaire Step3: The core group discussed the questionnaire and reserved items, and submitted the discuss results to the expert group up for review and determine the final reserved items
Method 2	Step1: Except for expert survey, items that are suggested to be deleted by any of the 7 indexes will not be retained Step2: For the items retained in the step 1, the top 3 items were selected according to the assignment value of the expert survey to form the simply questionnaire Step3: Same as step 3 of selecting method 1
Method 3	Step1: According to the 3 methods including expert survey, CTT and IRT, the top 3 items will be screened respectively Step2: The items that were selected in the top 3 most times by 3 methods will be selected to form the simply questionnaire Step3: Same as step 3 of selecting method 1
Method 4^a^	Step1: Except for expert survey, items that are suggested to be deleted by any of the 2 method will not be retained Step2: For the items retained in the step 1, the top 3 items were selected according to the assignment value of the expert survey to form the simply questionnaire Step3: Same as step 3 of selecting method 1

^a^Selecting method 4 is essentially the same as method 2, so the results of this method will not be listed repeatedly in this study

### Evaluation on short-form versions

#### Data sources

Evaluation was proceeded on the psychometric properties of short-form versions from CCMQ, after 3 short forms had been developed. (a) The data of the psychometric properties such as fasibility, validity and reliability, which were collected from teachers and students at 3 universities/colleges belonging to Beijing University of Chinese Medicine, Beihang University, Hebei University, Jiujiang University and Shandong University of Traditional Chinese Medicine. October to December 2020 as the collective periods and convenience sample method were selected in the research; and (b) the data of 21,948 participants were used for evaluationg the sensitivity and specificity of the new short-form CCMQs, which obtained from an epidemiological survey between December 2005 to January 2007 in China covering 9 provinces by purposive sample method[23, 39].

#### Inclusion criteria

Inclusion Criteria were as follows. (a) Ages 18 to 64 years old; and (b) took part in the survey voluntarily.

#### Exclusion criteria

Exclusion Criteria were as follows. (a) Having mental illness and/or conscious behavior disorder; (b) having severe chronic disease; (c) patients in the acute phase of chronic disease or suffering from an acute disease; (d) pregnant women or women within 6 months after childbirth; and (e) unable to understand the questions of CCMQ due to their own reasons and still unable to well understand after explanation and guidance. Participants meeting one or more criteria mentioned abovewere excluded.

#### Sample size calculation

Factor analysis is used to evaluate the construct validity of the scale, and the reliability of the evaluation results is closely related to the sample size. Generally, the ratio of the participants to the number of variables should be > 5:1, usually 5–10 times [54, 55]. Meanwhile, 20% of the fall-off and nonconforming samples were considered. A total of 13 basic information variables were set, and the item variables of the 3 short form versions were 27, 23, and 27, respectively. So, 225–500 samples should be collected for each short form scale and a total more than 725 samples.

#### Instruments of research

This research including a basic information table, 3 short-form versions of CCMQ, and SF-36 (in this study, the Chinese version of SF-36 developed by the Institute of Social Medicine and General Medicine, Zhejiang University) as a validity evaluation instrument [26, 56]. Then, data from the original CCMQ was used for evaluating the sensitivity and specificity.

#### Scoring algorithm

All the scoring algorithms for 3 short-form used the method of the standard of CACM-CDCTCM[21]. (a) Likert 5-level scoring method is adopted from 1 to 5; (b) raw score = add score value of all items; (c) derived score = 100*(raw score−total number of items)/(total number of items*4). And the derived score of each scale ranges from 0 to 100.

#### The cut-off value of the original CCMQ

The cut-off value was established in CDCTCM for the original CCMQ [21]. The cut-off value for GTC is 60 while the unbalanced BC types are 30(tendency) and 40(yes).

#### Evaluation indices

##### Psychometric property

First, the validity of scales evaluation including construct validity (used the method of Exploratory factor analysis, EFA), criterion validity (SF-36 was selected as a criterion tool), divergent validity (Obesity is an important feature of some BC types, and presents certain clustering characteristics. So, obesity is used for evaluating the divergent validity, while the cut-off point criterion to distinguish obesity is BMI = 25 [22]). Secondly, the reliability of scales evaluation including internal consistency reliability (Cronbach’s alpha coefficient should be > 0.7 in a total questionnaire, and in sub-scale should be > 0.6 but > 0.5 is also acceptable [43, 57]), test–retest reliability (a time interval of 2 weeks and test–retest coefficient > 0.6 were adopted, and preferably > 0.7 [26, 58]). Thirdly, the result data of evaluation indices were compared and the best of 3 short-forms were determined. In the EFA, (a) Kaiser–Meyer–Olkin(KMO) test and Bartlet test of Sphericity were used to evaluate whether the data were suitable for EFA; (b) the common factor whose characteristic value > 1 would be extracted through principal component analysis; and (c) The item whose factor loading would be evaluated as the cut-off criterion ≥ 0.4 through the maximum variance rotation method.

##### Diagnostic validity

The good psychometric property is very important for one scale instrument, however, as a new instrument, it is equally important to have a good diagnostic efficacy compared with the original instrument. So, the receiver operating characteristic (ROC) curve was used to evaluate the sensitivity and specificity of the short forms, and the area under the ROC curve (AUC) was calculated. (A) The cut-off scores 60 for GTC, and 40 and 30 for unbalanced BC types [21], were used for the ROC calculated. The original CCMQ served as the reference standard for ROC curves; (B) meanwhile, the item and data for a best questionnaire of 3 short-forms were extracted from the original CCMQ; (C) a dataset of 21,948 participants was analyzed, which conducted from December 2005 to January 2007 in China and covered 9 provinces as an epidemiological survey [23]. This data was adopted using the original CCMQ; and (D) then, the diagnostic utilities were evaluated by the AUC, and the sensitivity and specificity of the short-form were calculated. An AUC value is between 0.7 and 0.8 is considered acceptable, and an AUC value is ≥ 0.8 is considered excellent [59].

#### Quality control

In order to ensure the authenticity and accuracy of the data, the quality of the data is strictly controlled. (a) If no item data is missing in the scale of short-form, it is considered qualified. On the contrary, if the response data is missing ≥ 1 item, it is unqualified; (b) if responses had been chosen ≥ 1 in one item, it is unqualified; (c) if the respondent does not give informed, it is unqualified; (d) in criterion validity analysis, the scale SF-36 should have no missing data about responses. If the response data is missing ≥ 1 item, it is unqualified; (e) if apparently arbitrary, or items that are mutually verified are judged to be untrustworthy, it is unqualified; and (f) The aim is to evaluate the psychometric properties of the short-forms in this study, so participants were allowed to decide whether to fill in the information of other participants except for the items in the scale, to protect the privacy of participants and the response quality of scales.

### Statistical analyses

In the measurement data, the mean was $\overline{{\text{X}}}$  ± SD, and the independent sample T-test was used for mean comparison. Wilcoxon rank-sum test was used for data not subject to normal distribution and homogeneity of variance. Composition ratio was used for evaluating the distribution of experts and demographic characteristics of participants. The methods of CVCR, correlation coefficient, discrete tendency, internal consistency, and factor loading were used for the selection method of CTT. Discrimination parameters and Difficulty parameters were used for the selection method of IRT. KMO and Bartlett sphericity test were used for the unidimensionality test and applicability analysis of EFA method. The psychometric properties were analyzed by Construct validity, criterion validity, divergent validity, test–retest reliability and internal consistency reliability. The diagnostic validity was evaluated by AUC. Statistical software Multilog 7.03 was used for IRT and SPSS21.0 was used for other statistical analyses. *P* < 0.05 was considered statistically significant.

## Results

### Development of short-form

#### Selection result of expert survey 

In the result, 49 questionnaires including 41 e-questionnaires and 8 mannual-questionnaires were successfully collected. The demographic characteristics of the respondents (n = 49) are presented in Table 2.

**Table 2:** Demographic characteristics of the respondents (n = 49)

Demographic characteristics	Values
Sex (n %)	
Male	21 (42.86%)
Female	28 (57.14%)
Age (years, n %)	
30–39	10 (20.41%)
40–49	24 (48.98%)
50–59	13 (26.53%)
≥ 60	2 (4.08%)
Work experience (years, n %)	
< 10	6 (12.24%)
11–15	12 (24.49%)
16–20	8 (16.33%)
> 20	23 (46.94%)
Education (n %)	
Bachelor Degree	4 (8.16%)
Master Degree	9 (18.37%)
Doctor Degree	36 (73.47%)
Professional title level (n %)	
Professor/consultant	45 (91.84%)
Assistant professor/researcher/registrar	3 (6.12%)
Primary researcher/general practioner	1 (2.04%)
Occupation class^a^ (n %)	
Clinical	46 (93.88%)
Education	35 (71.00%)
Research	4 (8.00%)

^a^32 respondents belong both clinical and education, 4 respondents belong both clinical and research

The participants included 21 males and 28 females with a mean age of (46.92 ± 7.88) (range: 32–63 years old). Forty-three experts (87.76%) had over 10 years of experience in their professional areas, 91.84% experts had the master (18.37%) and doctoral degree (73.47%), and 91.84% experts had senior professional title (Professor/Consultant). So, the authority of the expert is also assured.

The response rate of e-questionnaire for strangers of experts was 16.27% in this study, which was sinilar and higher than the general (the response rate of strangers surveyed by E-mail is 16.15%) [60]. Meanwhile, mannual-questionnaire was 100% response rate. So, the questionnaire recovery rate of the two methods is reasonable and higher, which reflects the good positivity of the experts.

In addition, the broad representation of experts is also consideration dimension in our study. First, The panel consisted of experts from three related occupations including physician, scholar and researcher. Moreover,in addition to the views of the senior professional title experts, we also listened to the views of the intermediate(3 experts, 6.12%) and Primary(1 experts, 2.04%) experts to some extent (Table 2).

According to the scoring and selecting criteria, the weighted scores of each item were sorted. The top 3 items with the highest weighted score were retained in each BC type scale. A total of 26 items were retained for the simplified questionnaire (Q2 “Did you get tired easily?” is a common item of QDC and GTC) (Table 3).

**Table 3:** The select results

Scale	Item of original questionnaire (CCMQ)	ES^a^					CTT^b^					IRT						
		Point score of the 1st important item	Point score of the 2nd important item	Point score of the 3rd important item	Weighted total score	Select result	Criteria value-critical ratio method	Correlation coefficient method	Discrete tendency method	Internal consistency method	Factor loading	Discrimination parameters a	Difficulty parameters b				Retain/delete	Result of select
													b1	b2	b3	b4		
GTC	(1) Were you energetic?	40	6	2	134	#	134.718	0.483	1.072	0.612	0.401	0.29	− 5.12	− 1.72	1.1	4.51	×	
	(2) Did you get tired easily?		12	11	35	#	223.634#	0.637#	1.092	0.566#	0.714#	0.29	− 4.95	− 1.66	1.07	4.36	×	
	(7) Was your voice weak when talking?	1		5	8		198.387	0.597#	1.087	0.579#	0.646#	1.83	− 1.66	− 0.77	0.32	1.52	√	#
	(8) Did you feel in low spirits and depressed?		3	4	10		240.036#	0.651#	1.097	0.561#	0.730#	0.2	− 11.99	− 3.79	3.45	9.27	×	
	(21) Did you feel more vulnerable to the cold than others (winter coldness, air conditioners, fans, etc.)?	1	2	4	11		211.092#	0.578	1.381#	0.597	0.561	2.5	− 0.85	− 0.16	0.76	1.61	√	#
	(27) Did you forget things easily?		1	3	5		198.395	0.577	1.226	0.589	0.613	2.06	− 0.53	0.21	1.04	1.89	√	#
	(53) Could you adapt yourself to external natural or social environment changes?	6	21	4	64	#	67.788	0.253	1.450#	0.712	− 0.064	1.59	− 0.39	0.52	1.15	2.15	√	
	(54) Did you easily experience insomnia?		2	12	16		197.871	0.566	1.261#	0.594	0.576	1	− 0.69	0.72	2.22	3.68	×	
	Item X	1	2	4	11													
QDC	(2) Did you get tired easily?	37	4	5	124	#	185.378#	0.665#	1.092	0.717#	0.688#	0.3	− 4.84	− 1.52	1.28	4.67	×	
	(3) Did you experience shortness of breath?	3	12	10	43	#	183.936#	0.668#	1.124	0.716#	0.698#	0.28	− 5.09	− 1.69	1.12	4.53	×	
	(4) Did you get palpitations?		1	4	6		172.008#	0.650#	1.045	0.720#	0.686#	0.28	− 5.2	− 1.76	1.15	4.64	×	
	(5) Did you get dizzy easily or become dizzy when standing up?	4	1	2	16		155.373	0.598	1.129	0.732	0.604	0.27	− 8.71	− 4.1	1.83	7.94	×	
	(6) Did you prefer quietness and not like to talk?		6	3	15		167.522	0.615	1.236#	0.732	0.61	0.35	− 6.84	− 3.42	0.99	5.63	×	
	(7) Was your voice weak when talking?	4	10	6	38	#	167.474	0.64	1.087	0.722	0.658	1.91	− 1.71	− 0.79	0.28	1.43	√	#
	(22) Did you catch colds more easily than others?		11	6	28		122.611	0.52	1.160#	0.75	0.473	0.25	− 10.85	− 5.75	− 0.23	4.02	×	
	(26) Did you sweat easily when your physical activity increased slightly?	1	4	13	24		136.172	0.541	1.331#	0.755	0.47	2.21	− 0.78	− 0.12	0.69	1.56	√	#
YaDC	(17) Did your hands or feet feel cold or clammy?	20	10	4	84	#	216.94	0.621	1.345#	0.781	0.61	1.74	− 0.2	0.47	1.32	2.13	√	#
	(18) Did you feel cold easily in your abdomen, back, lower back or knees?	3	9	13	40	#	306.077#	0.732#	1.33	0.752#	0.746#	1.9	− 0.22	0.42	1.23	2.03	√	#
	(19) Were you sensitive to cold and tended to wear more clothes than others?	17	12	11	86	#	392.290#	0.798#	1.352#	0.733#	0.826#	0.27	− 10.69	− 7.14	− 3.2	− 0.12	×	
	(21) Did you feel more vulnerable to the cold than others (winter coldness, air conditioners, fans, etc.)?	7	5	7	38		377.600#	0.783#	1.381#	0.738#	0.808#	2.39	− 0.88	− 0.17	0.75	1.62	√	#
	(22) Did you catch colds more easily than others?	1	8	2	21		165.433	0.546	1.16	0.79	0.531	0.31	− 8.98	− 5.04	− 0.4	3.04	×	
	(52) Did you feel uncomfortable when you drank or ate something cold, or did you avoid to drinking or eating cold items?	1	3	8	17		237.549	0.666	1.324	0.769	0.649	1.48	− 0.37	0.57	1.16	2.13	√	
	(55) Did you easily contract diarrhea when you were exposed to cold or ate (or drank) something cold?			4	4		146.47	0.503	1.165	0.798	0.457	0.82	0.13	1.3	2.81	4.39	×	
YiDC	(16) Did the palms of your hands or soles of your feet feel hot?	24	11	7	101	#	167.297	0.547	1.183	0.677	0.54	1.59	− 1.04	− 0.35	0.66	1.74	√	#
	(20) Did your body and face feel hot?	6	10	6	44	#	191.512	0.614#	1.04	0.654#	0.654#	0.31	− 9.6	− 6.4	− 2.93	− 0.14	×	
	(29) Were your lips redder than in the past?		1	2	4		118.315	0.465	0.962	0.687	0.465	1.41	− 0.12	0.54	1.68	2.81	√	
	(35) Did your skin or lips feel dry?	8	5	5	39		235.236#	0.639#	1.243#	0.652#	0.636#	2.22	− 0.81	− 0.12	0.73	1.65	√	#
	(38) Did you experience hot flashes?	4	2	5	21		140.144	0.535	0.975	0.672	0.567	1.19	− 0.18	0.39	1.16	2.25	√	
	(44) Did your eyes feel dry and you used eye drops?		3	4	10		206.551#	0.603	1.21	0.662	0.597	1.18	− 0.14	0.73	2.01	3.22	×	
	(46) Did you often feel parched and need to drink water?	5	11	9	46	#	222.866#	0.621#	1.252#	0.658#	0.616#	1.5	− 0.61	− 0.02	0.54	1.45	√	#
	(57) Did you get constipated easily or have dry stools?	2	6	10	28		159.132	0.509	1.229#	0.69	0.456	1	0.54	1.58	2.79	3.98	×	
	Item X			1	1													
PDC	(13) Did you feel chest or abdominal stuffiness?	5	5	6	31		200.258	0.588	1.118	0.692	0.61	1.59	− 1.22	− 0.33	0.8	1.87	√	#
	(15) Did your body feel heavy or lethargic?	13	8	11	66	#	220.836#	0.617#	1.158	0.685#	0.638#	1.58	− 1.05	− 0.36	0.64	1.61	√	#
	(28) Did you have an excessively oily forehead and/or T− zone?	3	8	5	30		175.247	0.524	1.340#	0.718	0.458	1.33	− 0.88	0.55	1.64	2.61	√	
	(42) Did you have upper eyelid swelling?				0		150.838	0.527	1.034	0.703	0.535	1.67	− 0.48	0.03	0.61	1.5	√	
	(49) Did your mouth feel sticky?	4	6	10	34		211.805#	0.647#	1.096	0.677#	0.681#	0.23	− 9.02	− 4.3	− 1.24	1.93	×	
	(50) Was your abdomen flabby?	9	3	4	37	#	219.403#	0.611#	1.366#	0.695	0.582	1.48	− 0.36	0.57	1.16	2.12	√	
	(51) Did you have an abundance of phlegm, especially in your throat?	3	11	5	36	#	183.603	0.573	1.212#	0.698	0.567	1.59	− 0.38	0.53	1.15	2.14	√	#
	(58) Did your tongue have a thick coating?	1	8	8	27		201.968	0.599	1.172	0.690#	0.612#	1.2	0.36	1.26	2.37	3.41	×	
	Item X	1			3													
DHC	(39) Did your nose or your face feel greasy, oily, or shiny?	9	6	9	48		229.262#	0.633#	1.353#	0.632	0.602	1.22	− 0.45	0.16	0.96	2.07	√	#
	(41) Did you get acne or sores easily?	10	7	7	51	#	168.292	0.583	1.142	0.636	0.573	1.65	− 0.62	− 0.06	0.65	1.62	√	#
	(48) Did you have a bitter or strange taste in your mouth?	15	4	3	56	#	196.26	0.596	1.218#	0.637	0.588	0.21	− 8.79	− 3.85	− 0.9	2.21	×	
	(56) Did you pass sticky stools and/or feel that your bowel movement was incomplete?	12	14	11	75	#	206.904	0.614	1.244#	0.630#	0.607#	0.98	− 0.02	1.04	3.28	3.64	×	
	(59) Did your urethral canal feel hot when you urinated, or did your urine have a dark color?	2	14	6	40		208.849#	0.630#	1.131	0.616#	0.661#	0.99	− 1.22	− 0.23	0.7	2.08	×	
	(60) Was your vaginal discharge yellowish (only for female interviewees)? /Was your scrotum always wet (only for male interviewees)?	1	4	13	24		209.022#	0.633#	1.168	0.616#	0.657#	1.1	− 1.02	− 0.09	0.8	2.14	√	#
BSC	(27) Did you forget things easily?	1	13	4	33		172.811	0.576	1.226	0.659	0.557	1.88	− 0.51	0.24	1.12	1.96	√	#
	(33) Did black or purple bruises appear on your skin for no reason?	7	10	17	58	#	138.191	0.532	0.97	0.66	0.555	2.06	− 0.65	0.11	0.99	1.92	√	#
	(36) Did you have visible capillary (thread) veins on your cheeks?	2	6	1	19		135.907	0.519	1.051	0.667	0.521	0.91	− 0.11	0.74	1.78	3.07	×	
	(37) Did you feel pain somewhere in your body?	6	9	7	43		181.312#	0.589#	1.231#	0.655#	0.575#	1.05	0.02	0.86	1.82	3.02	×	
	(40) Did you have a dark face or get brown spots easily?	19	6	5	74	#	225.649#	0.692#	1.308#	0.618#	0.706#	1.58	− 0.56	− 0.04	0.65	1.57	√	
	(43) Did you get dark circles under the eyes easily?	2	2	4	14		173.028	0.584	1.238#	0.657	0.573	1.7	− 0.53	0.02	0.64	1.53	√	#
	(45) Were your lips darker, more blue or purple than usual?	11	3	7	46	#	188.950#	0.620#	1.223	0.643#	0.626#	1.34	− 0.15	0.71	1.88	2.95	√	
	Item X	1		4	7													
QSC	(8) Did you feel in low spirit sand depressed?	26	3	7	91	#	292.869#	0.753#	1.097	0.734#	0.789#	0.26	− 8.79	− 2.69	2.83	7.48	×	
	(9) Did you easily feel anxious and worried?	10	11	7	59	#	302.992#	0.763#	1.105	0.732#	0.798#	1.88	− 0.52	0.2	1.08	1.93	√	#
	(10) Did you feel overly sensitive, vulnerable or emotionally upset?	4	16	9	53	#	309.439#	0.753#	1.187#	0.735#	0.782#	1.83	− 0.55	0.18	1.12	2.09	√	
	(11) Were you easily scared or frightened?		3	2	8		237.877	0.682	1.113#	0.753	0.695	1.93	− 0.57	0.21	1.28	2.07	√	#
	(12) Did you experience distention in the underarm or breast?	5	5	10	35		157.163	0.534	1.089	0.786	0.489	1.99	− 0.59	0.2	1.26	2.17	√	#
	(14) Did you sigh without reason?	3	8	9	34		242.679	0.684	1.113	0.752	0.691	1.53	− 1.22	− 0.26	0.9	2.1	√	
	(47) Did your throat feel strange (i.e., as if something was stuck or there was a lump in your throat)?	1	3	5	14		145.116	0.485	1.262#	0.808	0.376	1.6	− 0.6	0.01	0.59	1.46	√	
SDC	(23) Did you sneeze even when you did not have a cold?	7	6	5	38		235.553#	0.627	1.047	0.708#	0.612	2.4	− 0.78	− 0.08	0.84	1.66	√	#
	(24) Did you have a runny or stuffy nose even when you did not have a cold?	8	5	6	40		219.075#	0.63#	1.081#	0.709	0.613	2.59	− 0.75	− 0.07	0.79	1.64	√	#
	(25) Did you cough due to seasonal changes, temperature changes or unpleasant odors?	6	8	12	46	#	166.306	0.568	0.948	0.72	0.564	2.04	0.84	− 0.15	0.7	1.53	√	
	(30) Did you have allergies? (E.g. medicine, food, odors, pollen, pet dander or during seasonal or weather change etc.)	25	14	4	107	#	196.032	0.674#	1.089#	0.695#	0.687#	1.71	− 0.56	0.14	1.14	2.12	√	
	(31) Did you get hives/urticaria easily?	2	13	15	47	#	190.712	0.675#	1.036	0.693#	0.696#	1.81	− 0.58	0.12	1.13	2.18	√	
	(32) Did your skin have purpura (purple spots, ecchymosis) due to allergies?			1	1		133.321	0.587	0.811	0.712	0.625#	1.95	− 0.68	0.1	0.96	1.86	√	
	(34) Did your skin turn red and show traces when you scratched it?		2	5	9		228.274#	0.622	1.212#	0.721	0.589	2.08	− 0.82	− 0.13	0.73	1.65	√	#
	Item X	1	1	1	6													

Abbreviations: CCMQ: constitution in Chinese medicine questionnaire; GTC: Gentleness constitution, QDC: Qi-deficiency constitution, YaDC: Yang-deficiency constitution,YiDC: Yin-deficiency constitution, PDC: phlegm-dampness constitution, DHC: damp-heat constitution, BSC: blood-stasis constitution, QSC: Qi-stagnation constitution and ISC: inherited-special constitution; ES: expert survey; CTT: Classical test theory; IRT: Item response theory

^a^.the item score, means for example, item (1) Were you energetic? was considered by 40 experts to be the first important item on the Gentleness body constitution type scale, and scored 40 point value; ^b^.the value of Criteria value-critical ratio method is the t-value of the independent sample t-test, the value of Correlation coefficient method is the correlation coefficient of item-scale score, the value of discrete tendency method is the SD of the mean item score, the internal consistency method is Cronbach’s alpha coefficient of body constitution scale after deleting items, the factor loading method is the factor loading value of the item in the body constitution scale belonged; Item X. the item that need to be added according to the experts opinion; #: the item that should be retained based on result of ES, CTT, or IRT; × : recommend to delete based on result of IRT; √: recommend to retain based on result of IRT

#### Selection result of CTT

Five indices of the CTT method were analyzed, with the top 3 items of every index in each BC type scale selected and retained to construct a short-form questionnaire (Table 3). (a) CVCR method. The independent sample t test results of each item indicated that the *P* < 0.05 and t > 3.000 in all items, and the discrimination of all items was well; (b) correlation coefficient method. Except for item 53 of the GTC scale (Could you adapt yourself to external natural or social environment changes? and *r* =.253), Pearson correlation coefficients of the item-scale were *r* > 0.4, which had a good correlation with the BC type scale which the items belonged; (c) discrete tendency method. The results of each item indicated that there are 5 items have SD values < 1.00 and should be deleted. The 5 items include items 29 and 38 in YiDC scale, item 33 in BSC scale, and items 25 and 32 in SDC scale, respectively; (d) internal consistency method. The Cronbach’s alpha coefficient results indicated that item 53 reduced the internal consistency coefficient of the GTC scale and item 55 reduced the internal consistency coefficient of the YaDC scale; and (e) Factor loading method. The exploratory factor analysis indicated that item 53 had a factor loading *r* = − 0.064 in the GTC scale and item 47 had a factor loading *r* = 0.376 in the QSC scale, and both the absolute values were < 0.4.

#### Selection result of IRT

A) Unidimensionality test. The adaptability analysis of EFA showed that KMO = 0.943 and Bartlett sphericity test χ2 = 1,724,483.085, df = 1770, *P* < 0.001. Thus, the data in the present study are suitable for EFA. Meanwhile, the unidimensional hypothesis test results using EFA showed that the first and second characteristic values of CCMQ are 12.545 and 2.961, and the ratio of the first and second characteristic values is > 3, indicating that it conforms to the unidimensional construct and can be analyzed by IRT. b) Discrimination parameters a. In total, 12 items had discrimination value < 0.5 and included 3 items (items 1, 2 and 8) in the GTC scale, 6 items (items 2, 3, 4, 5, 6 and 22) in the QDC scale, 2 items (items 19 and 22) in the YaDC scale, 1 item (item 20) in the YiDC scale, 1 item (item 49) in the PDC scale, 1 item (item 48) in the DHC scale, and 1 item (item 8) in the QSC scale. c) Difficulty parameters b. Totally, 18 items with a difficulty value > 3 include 4 items (items 1, 2, 8 and 54) in the GTC scale, 6 items (items 2, 3, 4, 5, 6 and 22) in the QDC scale, 3 items (items 19, 22 and 55) in the YaDC scale, 3 items (items 20, 44, and 57) in the YiDC scale, 1 item (item 58) in the PDC scale, 1 item (item 56) in the DHC scale, 2 items (items 36 and 37) in the BSC scale, and 1 item (item 8) in the QSC scale. d) A total of 21 items were deleted based on the results of discrimination and difficulty parameter. In the remaining items, the 3 items with the best discrimination and difficulty parameters were reserved, and 23 items were reserved to construct a simplified questionnaire (Table 3).

#### Short-form version of selecting method 1

By using the first method to simplify CCMQ, 27 items are retained, including 4 items for the GTC, DHC and SDC scales, and 3 items for the other BC type scales. As the original CCMQ, item 2 is the constituent item in the GTC and QDC scales, item 8 is the constituent item in the GTC and QSC scales, item 21 is the constituent item in the GTC and YaDC scales.

#### Short-form version of selecting method 2

By using the second method to simplify CCMQ, 23 items are retained, all BC type scales contain 3 items except for the QDC scale that contains 2 items. As the original CCMQ, item 7 is the constituent item in the GTC and QDC scales, item 21 is the constituent item in the GTC and YaDC scales, item 27 is the constituent item in the GTC and BSC scales.

#### Short-form version of selecting method 3

By using the third method to simplify CCMQ, 27 items are retained. In the scales of each BC type scale, the GTC scale contains 2 items, the scales of QDC, PDC, BSC, QSC, and SDC all contain 3 items, and the scales of YaDC, YiDC and DHC all contain 4 items. Items 2 and 7 are the constituent items in the GTC and QDC scales.

#### Core group and expert group discussion and verification

The retained items from the 3 methods were discussed by the core group and the expert group. In the edition that was simplified by the first method, item 7 is deleted, and item 1 is reinstated as a component of the GTC scale. The item in the editions simplified by the second and third methods were not adjusted. Finally, 3 simplified questionnaires of CCMQ (27, 23 and 27 items) are developed, which are named as WangQi Nine body constitution questionnaire of Traditional Chinese Medicine (Short-form) (SF-WQ9CCMQ)—A, B, and C.

#### Short-forms of CCMQ

SF-W9CCMQ -A include 27 items, and most BC type scales in this edition retain 3 items as expected and 4 in the GTC, DHC and SDC scales. SF-W9CCMQ -B and SF-W9CCMQ -C include 23 and 27 items respectively and most BC type scales retain 3 items in both. The difference is that QDC scale of SF-W9CCMQ -B has only 2 items retained, whereas 2 items in GTC and 4 items in YaDC, YiDC and DHC are retained in SF-W9CCMQ -C.

### Evaluation of short-form

#### Demographic characteristics of participants

A total of 380 cases who met the inclusion criteria participated in the survey of SF-WQ9CCMQ- A, 384 cases participated in the survey of SF-WQ9CCMQ- B and 349 cases participated in the survey of SF-WQ9CCMQ- C, respectively. The detailed information about the results of socio-demographic characteristics of samples were showed in Table 4.

**Table 4:** Demographic characteristics^a^

SF-WQ9CCMQ- Editions A^b^		SF-WQ9CCMQ- Editions B^c^		SF-WQ9CCMQ- Editions C^d^	
Demographic characteristics	Values	Demographic characteristics	Values	Demographic characteristics	Values
Gender (n %)		Gender (n %)		Gender (n %)	
Male	147 (38.79%)	Male	140 (36.55%)	Male	127 (36.49%)
Female	232 (61.21%)	Female	243 (63.45%)	Female	221 (63.51%)
Age (years, n %)		Age (years, n %)		Age (years, n %)	
18–20	193 (48.61%)	18–20	255 (67.28%)	18–20	224 (64.74%)
21–25	158 (41.69%)	21–25	107 (28.23%)	21–25	110 (31.79%)
> 25	28 (7.39%)	> 25	17 (4.49%)	> 25	12 (3.47%)
Education (n %)		Education (n %)		Education (n %)	
Below Bachelor Degree	2 (0.53%)	Below bachelor degree	8 (2.12%)	Below bachelor degree	33 (9.68%)
Bachelor Degree	317 (84.08%)	Bachelor degree	326 (86.47%)	Bachelor degree	270 (79.18%)
Master and Doctor Degree	58 (15.38%)	Master and doctor degree	43 (15.38%)	Master and doctor degree	38 (11.14%)
Height (n %)		Height (n %)		Height (n %)	
< 150	1 (0.26%)				
150 ~	63 (16.76%)	150 ~	80 (20.89%)	150 ~	64 (18.66%)
160 ~	157 (41.76%)	160 ~	155 (16.97%)	160 ~	151 (44.02%)
170~	117 (31.12%)	170 ~	112 (40.47%)	170 ~	87 (25.36%)
180 ~	38 (10.11%)	180 ~	36 (9.40%)	180 ~	41 (11.95%)
Weight (n %)		Weight (n %)		Weight (n %)	
< 40	2 (0.54%)	< 40	2 (0.52%)		
40 ~	82 (21.98%)	40 ~	83 (21.73%)	40 ~	72 (21.24%)
50 ~	126 (33.78%)	50 ~	134 (35.08%)	50 ~	123 (36.28%)
60 ~	76 (20.38%)	60 ~	84 (21.99%)	60 ~	74 (21.83%)
70 ~	52 (13.94%)	70 ~	41 (10.73%)	70 ~	40 (11.80%)
80 ~	22 (5.90%)	80 ~	22 (5.76%)	80 ~	18 (5.31%)
90 ~	8 (2.14%)	90 ~	13 (3.40%)	90 ~	7 (2.06%)
100 ~	5 (1.34%)	100 ~	3 (1.85%)	100 ~	5 (1.47%)

^a^In order to proctect the privacy of participants and the quality of scales in this research, participants were allowed to dicide whether to fill in the information of other participants except the items in the scale; ^b^379 participants provide the data of gender and age, 377 of education, 376 of height, and 373 of weight; ^c^383 participants provide the data of gender and height, 379 of age, 377 of education, and 382 of weight; ^d^348 participants provide the data of gender, 346 of age, 341 of education, 343 of height, and 339 of weight

#### Conform to the quality control scale

The quality of the SF-WQ9CCMQ- A, B, and C were evaluated and screened according to the quality control standard.The detail was showed 338, 372 and 340 cases were used for validity and reliability evaluation, and 82, 120, and 106 cases were used for test–retest reliabiliy evaluation, for the 3 short-forms respectively. SF-WQ9CCMQ- A, B, C have the mean of completion time is 4.23 ± 3.492 min (1 to 25 min), 3.01 ± 2.828 min (1–30 min), and 4.00 ± 3.704 min (1–30 min).

#### Validity evaluation

##### Construct validity

All KMO values of 3 short-forms > 0.8, and Bartlett Test of Sphericity results well, so the data of 3 short-forms were feasible for EFA. Both 8 common factors were extracted in SF-WQ9CCMQ- A, and B, and 7 common factors in SF-WQ9CCMQ- C. The accumulated coutribution rates were 61.294%, 59.360% and 59.484%.The detailed results about the 3 total scales and BC type scales were in Tables 5 and 6.

**Table 5:** The results of KMOand Bartlett test of sphericity

Edition of short-form	KMO value	Bartlett test of sphericity		
		Χ^2^	df	P -value
A	0.847	2809.382	351	0.000
B	0.860	2260.698	253	0.000
C	0.832	2556.735	351	0.000

Abbreviations: KMO value = Kaiser–Meyer–Olkin value

**Table 6:** Common factors and total variance explained of 3 short-forms

SF-WQ9CCMQ- Editions A	Component	Initial Eigenvalues			Extraction Sums of Quared Loadings			SF-WQ9CCMQ- Editions B	Component	Initial Eigenvalues			Extraction Sums of Quared Loadings		
		Total	% of Variance	Cumulative %	Total	% of Variance	Cumulative %			Total	% of Variance	Cumulative %	Total	% of Variance	Cumulative %
Total	1	6.697	24.803	24.803	6.697	24.803	24.803	Total	1	5.862	25.485	25.485	5.862	25.485	25.485
	2	2.125	7.872	32.674	2.125	7.872	32.674		2	1.712	7.445	32.930	1.712	7.445	32.930
	3	1.705	6.316	38.990	1.705	6.316	38.990		3	1.517	6.597	39.528	1.517	6.597	39.528
	4	1.507	5.583	44.573	1.507	5.583	44.573		4	1.334	5.800	45.328	1.334	5.800	45.328
	5	1.269	4.699	49.272	1.269	4.699	49.272		5	1.125	4.891	50.219	1.125	4.891	50.219
	6	1.124	4.163	53.435	1.124	4.163	53.435		6	1.101	4.786	55.005	1.101	4.786	55.005
	7	1.064	3.942	57.377	1.064	3.942	57.377		7	1.002	4.356	59.360	1.002	4.356	59.360
	8	1.057	3.916	61.294	1.057	3.916	61.294		8	0.904	3.929	63.289			
	9	0.923	3.418	64.711					9	0.882	3.834	67.123			
	10	0.863	3.197	67.908					10	0.764	3.320	70.443			
	11	0.820	3.037	70.945					11	0.725	3.154	73.597			
	12	0.742	2.747	73.693					12	0.703	3.057	76.654			
	13	0.707	2.618	76.311					13	0.694	3.015	79.669			
	14	0.645	2.390	78.701					14	0.652	2.837	82.506			
	15	0.611	2.263	80.964					15	0.625	2.718	85.224			
	16	0.597	2.213	83.177					16	0.563	2.448	87.672			
	17	0.565	2.093	85.269					17	0.530	2.303	89.975			
	18	0.546	2.021	87.291					18	0.484	2.105	92.080			
	19	0.516	1.909	89.200					19	0.430	1.869	93.949			
	20	0.484	1.792	90.992					20	0.408	1.772	95.722			
	21	0.450	1.667	92.659					21	0.363	1.580	97.301			
	22	0.411	1.523	94.182					22	0.321	1.396	98.697			
	23	0.396	1.468	95.650					23	0.300	1.303	100.000			
	24	0.338	1.253	96.903				GTC	1	1.476	49.194	49.194	1.476	49.194	49.194
	25	0.321	1.190	98.093					2	0.783	26.106	75.300			
	26	0.303	1.122	99.216					3	0.741	24.700	100.000			
	27	0.212	0.784	100.000				QDC	1	1.200	60.022	60.022	1.200	60.022	60.022
GTC	1	2.111	52.768	52.768	2.111	52.768	52.768		2	0.800	39.978	100.000			
	2	0.809	20.218	72.985				YaDC	1	2.003	66.783	66.783	2.003	66.783	66.783
	3	0.694	17.338	90.323					2	0.574	19.118	85.901			
	4	0.387	9.677	100.000					3	0.423	14.099	100.000			
QDC	1	1.726	57.542	57.542	1.726	57.542	57.542	YiDC	1	1.535	51.179	51.179	1.535	51.179	51.179
	2	0.710	23.668	81.211					2	0.842	28.067	79.245			
	3	0.564	18.789	100.000					3	0.623	20.755	100.000			
YaDC	1	2.158	71.923	71.923	2.158	71.923	71.923	PDC	1	1.515	50.506	50.506	1.515	50.506	50.506
	2	0.574	19.129	91.052					2	0.806	26.868	77.374			
	3	0.268	8.948	100.000					3	0.679	22.626	100.000			
YiDC	1	1.565	52.182	52.182	1.565	52.182	52.182	DHC	1	1.535	51.153	51.153	1.535	51.153	51.153
	2	0.844	28.131	80.313					2	0.834	27.802	78.955			
	3	0.591	19.687	100.000					3	0.631	21.045	100.000			
PDC	1	1.774	59.148	59.148	1.774	59.148	59.148	BSC	1	1.529	50.979	50.979	1.529	50.979	50.979
	2	0.631	21.031	80.180					2	0.774	25.790	76.769			
	3	0.595	19.820	100.000					3	0.697	23.231	100.000			
DHC	1	2.047	51.180	51.180	2.047	51.180	51.180	QSC	1	2.241	74.693	74.693	2.241	74.693	74.693
	2	0.744	18.600	69.780					2	0.413	13.764	88.457			
	3	0.673	16.834	86.615					3	0.346	11.543	100.000			
	4	0.535	13.385	100.000				SDC	1	1.595	53.177	53.177	1.595	53.177	53.177
BSC	1	1.570	52.340	52.340	1.570	52.340	52.340		2	0.913	30.436	83.612			
	2	0.909	30.311	82.650					3	0.492	16.388	100.000			
	3	0.520	17.350	100.000											
QSC	1	2.141	71.352	71.352	2.141	71.352	71.352								
	2	0.458	15.279	86.631											
	3	0.401	13.369	100.000											
SDC	1	1.904	47.596	47.596	1.904	47.596	47.596								
	2	0.989	24.734	72.330											
	3	0.731	18.282	90.612											
	4	0.376	9.388	100.000											

SF-WQ9CCMQ- Editions C	Component	Initial Eigenvalues			Extraction Sums of Quared Loadings		
		Total	% of Variance	Cumulative %	Total	% of Variance	Cumulative %
Total	1	6.166	22.837	22.837	6.166	22.837	22.837
	2	2.078	7.696	30.533	2.078	7.696	30.533
	3	1.696	6.281	36.813	1.696	6.281	36.813
	4	1.434	5.310	42.123	1.434	5.310	42.123
	5	1.367	5.064	47.188	1.367	5.064	47.188
	6	1.212	4.491	51.678	1.212	4.491	51.678
	7	1.094	4.053	55.731	1.094	4.053	55.731
	8	1.013	3.753	59.484	1.013	3.753	59.484
	9	0.909	3.366	62.850			
	10	0.826	3.060	65.910			
	11	0.797	2.950	68.860			
	12	0.749	2.774	71.634			
	13	0.743	2.753	74.387			
	14	0.739	2.736	77.124			
	15	0.680	2.519	79.643			
	16	0.670	2.481	82.123			
	17	0.637	2.361	84.484			
	18	0.592	2.192	86.676			
	19	0.547	2.024	88.700			
	20	0.540	1.998	90.699			
	21	0.504	1.866	92.564			
	22	0.417	1.546	94.110			
	23	0.388	1.438	95.548			
	24	0.370	1.371	96.918			
	25	0.339	1.255	98.174			
	26	0.300	1.111	99.285			
	27	0.193	0.715	100.000			
GTC	1	1.302	65.116	65.116	1.302	65.116	65.116
	2	0.698	34.884	100.000			
QDC	1	1.639	54.623	54.623	1.639	54.623	54.623
	2	0.707	23.580	78.204			
	3	0.654	21.796	100.000			
YaDC	1	2.530	63.252	63.252	2.530	63.252	63.252
	2	0.729	18.228	81.480			
	3	0.511	12.779	94.259			
	4	0.230	5.741	100.000			
YiDC	1	1.914	47.848	47.848	1.914	47.848	47.848
	2	1.000	25.006	72.854	1.000	25.006	72.854
	3	0.562	14.041	86.895			
	4	0.524	13.105	100.000			
PDC	1	1.512	50.404	50.404	1.512	50.404	50.404
	2	0.799	26.623	77.026			
	3	0.689	22.974	100.000			
DHC	1	1.811	45.274	45.274	1.811	45.274	45.274
	2	0.863	21.583	66.856			
	3	0.728	18.198	85.054			
	4	0.598	14.946	100.000			
BSC	1	1.367	45.564	45.564	1.367	45.564	45.564
	2	0.928	30.939	76.503			
	3	0.705	23.497	100.000			
QSC	1	2.207	73.555	73.555	2.207	73.555	73.555
	2	0.462	15.405	88.960			
	3	0.331	11.040	100.000			
SDC	1	1.628	54.276	54.276	1.628	54.276	54.276
	2	0.813	27.093	81.369			
	3	0.559	18.631	100.000			

Abbreviations: GTC: Gentleness constitution, QDC: Qi-deficiency constitution, YaDC: Yang-deficiency constitution,YiDC: Yin-deficiency constitution, PDC: phlegm-dampness constitution, DHC: damp-heat constitution, BSC: blood-stasis constitution, QSC: Qi-stagnation constitution and ISC: inherited-special constitution

The EFA results of 3 short-forms show that the cumulative variance explained of total scale of SF-WQ9CCMQ- A is best, and all the BC type scales are > 50% except SDC, and its comprehensive results are better than SF-WQ9CCMQ- B and C.

##### Criterion validity

In SF-WQ9CCMQ- A, Spearman correlation coefficients of GTC and dimensions of SF-36 were all positive except health transition (HT). Other BC type scales showed the opposite results in correlation. The abusolute value of Spearman correlation coefficient between BC types scores in SF-WQ9CCMQ- A and Physical component summary(PCS) and Mental component summary(MCS) in SF-36 is between 0.212 and 0.596(P < 0.01). GTC scores was positively correlated with the score of General health perceptions(GH) with the Spearman correlation coefficient was 0.433(P < 0.01). And there was a negative correlation between other BC types scores and the score of GH with the absolut value of the Spearman correlation coefficient was 0.218–0.346(P < 0.01).But the difference is that both SF-WQ9CCMQ- B and C do not show a good regular correlation as between A and SF36, and the detail was showd in Table 7.

**Table 7:** The results of the criterion validity of ecah BC types score and each dimension score of SF-36

Edition of short-form	BC type		PF	RP	BP	GH	VT	SF	RE	MH	HT	PCS	MCS
A	GTC	R	0.398^a^	0.371^a^	0.369^a^	0.469^a^	0.396^a^	0.222^a^	0.293^a^	0.286^a^	− 0.192^a^	0.565^a^	0.506^a^
		P	0.000	0.000	0.000	0.000	0.000	0.000	0.000	0.000	0.001	0.000	0.000
		N	319	319	319	319	319	319	319	319	319	319	319
	QDC	R	− 0.374^a^	− 0.318^a^	− 0.315^a^	− 0.381^a^	− 0.341^a^	− 0.159^a^	− 0.294^a^	− 0.249^a^	0.207^a^	− 0.485^a^	− 0.435^a^
		P	0.000	0.000	0.000	0.000	0.000	0.004	0.000	0.000	0.000	0.000	0.000
		N	319	319	319	319	319	319	319	319	319	319	319
	YaDC	R	− 0.313^a^	− 0.317^a^	− 0.296^a^	− 0.276^a^	− 0.174^a^	− 0.165^a^	− 0.296^a^	− 0.216^a^	0.063	− 0.431^a^	− 0.340^a^
		P	0.000	0.000	0.000	0.000	0.002	0.003	0.000	0.000	0.262	0.000	0.000
		N	319	319	319	319	319	319	319	319	319	319	319
	YiDC	R	− 0.206^a^	− 0.196^a^	− 0.328^a^	− 0.269^a^	0.187^a^	− 0.065	− 0.243^a^	− 0.164^a^	0.075	− 0.382^a^	− 0.254^a^
		P	0.000	0.000	0.000	0.000	0.001	0.249	0.000	0.003	0.179	0.000	0.000
		N	319	319	319	319	319	319	319	319	319	319	319
	PDC	R	− 0.364^a^	− 0.273^a^	− 0.263^a^	− 0.345^a^	− 0.264^a^	− 0.055	− 0.266^a^	− 0.084	0.204^a^	− 0.437^a^	− 0.284^a^
		P	0.000	0.000	0.000	0.000	0.000	0.330	0.000	0.137	0.000	0.000	0.000
		N	319	319	319	319	319	319	319	319	319	319	319
	DHC	R	− 0.186^a^	− 0.270^a^	− 0.317^a^	− 0.291^a^	− 0.200^a^	− 0.108	− 0.236^a^	− 0.165^a^	0.184^a^	− 0.378^a^	− 0.280^a^
		P	0.001	0.000	0.000	0.000	0.000	0.054	0.000	0.003	0.001	0.000	0.000
		N	319	319	319	319	319	319	319	319	319	319	319
	BSC	R	− 0.268^a^	− 0.276^a^	− 0.367^a^	− 0.335^a^	− 0.179^a^	− 0.072	− 0.288^a^	− 0.126^b^	0.053	− 0.447^a^	− 0.253^a^
		P	0.000	0.000	0.000	0.000	0.001	0.200	0.000	0.025	0.346	0.000	0.000
		N	319	319	319	319	319	319	319	319	319	319	319
	QSC	R	− 0.277^a^	− 0.302^a^	− 0.257^a^	− 0.299^a^	− 0.417^a^	− 0.111^b^	− 0.371^a^	− 0.427^a^	0.162^a^	0.409^a^	− 0.582^a^
		P	0.000	0.000	0.000	0.000	0.000	0.047	0.000	0.000	0.004	0.000	0.000
		N	319	319	319	319	319	319	319	319	319	319	319
	SDC	R	− 0.193^a^	− 0.180^a^	− 0.231^a^	− 0.252^a^	− 0.138^b^	− 0.116^b^	− 0.219^a^	− 0.052	0.061	− 0.315^a^	− 0.187^a^
		P	0.001	0.001	0.000	0.000	0.014	0.039	0.000	0.357	0.280	0.000	0.001
		N	319	319	319	319	319	319	319	319	319	319	319
B	GTC	R	0.306^a^	0.266^a^	− 0.157^a^	0.032	0.141^a^	− 0.051	0.199^a^	0.132^b^	− 0.142^a^	0.100	0.185^a^
		P	0.000	0.000	0.003	0.546	0.008	0.337	0.000	0.013	0.008	0.059	0.000
		N	355	355	355	355	355	355	355	355	355	355	355
	QDC	R	− 0.282^a^	− 0.256^a^	0.126^b^	− 0.015	− 0.053	0.046	− 0.212^a^	− 0.180^a^	0.096	− 0.077	− 0.163^a^
		P	0.000	0.000	0.017	0.779	0.323	0.390	0.000	0.001	0.072	0.147	0.002
		N	355	355	355	355	355	355	355	355	355	355	355
	YaDC	R	− 0.283^a^	− 0.219^a^	0.249^a^	0.048	− 0.070	0.185^a^	− 0.071	− 0.134^b^	0.118^b^	0.001	− 0.037
		P	0.000	0.000	0.000	0.367	0.187	0.000	0.183	0.011	0.026	0.981	0.491
		N	355	355	355	355	355	355	355	355	355	355	355
	YiDC	R	− 0.213^a^	− 0.213^a^	0.228^a^	0.018	− 0.111^b^	0.149^a^	− 0.166^a^	− 0.107^b^	0.164^a^	0.056	− 0.083
		P	0.000	0.000	0.000	0.734	0.037	0.005	0.002	0.044	0.002	0.292	0.119
		N	355	355	355	355	355	355	355	355	355	355	355
	PDC	R	− 0.272^a^	− 0.242^a^	0.172^a^	− 0.043	− 0.173^a^	0.051	− 0.196^a^	− 0.179^a^	0.181^a^	− 0.062	− 0.210^a^
		P	0.000	0.000	0.001	0.417	0.001	0.338	0.000	0.001	0.001	0.244	0.000
		N	355	355	355	355	355	355	355	355	355	355	355
	DHC	R	− 0.161^a^	− 0.129^b^	0.200^a^	0.050	− 0.079	0.111^b^	− 0.165^a^	− 0.093	0.195^a^	0.069	− 0.085
		P	0.002	0.015	0.000	0.352	0.137	0.036	0.002	0.079	0.000	0.198	0.111
		N	355	355	355	355	355	355	355	355	355	355	355
	BSC	R	− 0.226^a^	− 0.150^a^	0.182^a^	− 0.047	− 0.066	0.056	− 0.200^a^	− 0.147^a^	0.219^a^	− 0.001	− 0.140^a^
		P	0.000	0.005	0.001	0.380	0.216	0.294	0.000	0.006	0.000	0.985	0.008
		N	355	355	355	355	355	355	355	355	355	355	355
	QSC	R	− 0.209^a^	− 0.231^a^	0.200^a^	0.043	− 0.216^a^	0.113^b^	− 0.371^a^	− 0.362^a^	0.198^a^	0.042	− 0.336^a^
		P	0.000	0.000	0.000	0.419	0.000	0.033	0.000	0.000	0.000	0.434	0.000
		N	355	355	355	355	355	355	355	355	355	355	355
	SDC	R	− 0.108^b^	− 0.217^a^	0.244^a^	0.091	− 0.054	0.149^a^	− 0.150^a^	− 0.059	0.075	0.110^b^	− 0.023
		P	0.041	0.000	0.000	0.086	0.312	0.005	0.005	0.266	0.161	0.038	0.669
		N	355	355	355	355	355	355	355	355	355	355	355
C	GTC	R	0.271^a^	0.293^a^	− 0.074	0.103	0.178^a^	− 0.028	0.230^a^	0.099	− 0.138^b^	0.141^b^	0.180^a^
		P	0.000	0.000	0.179	0.063	0.001	0.614	0.000	0.073	0.012	0.010	0.001
		N	330	330	330	330	330	330	330	330	330	330	330
	QDC	R	− 0.310^a^	− 0.308^a^	0.119^b^	− 0.083	− 0.140^b^	0.051	− 0.241^a^	− 0.113^b^	0.126^b^	− 0.123^b^	− 0.155^a^
		P	0.000	0.000	0.030	0.131	0.011	0.352	0.000	0.040	0.022	0.026	0.005
		N	330	330	330	330	330	330	330	330	330	330	330
	YaDC	R	− 0.220^a^	− 0.158^a^	0.161^a^	− 0.110^b^	− 0.042	0.095	− 0.07	− 0.158^a^	0.012	− 0.026	− 0.068
		P	0.000	0.004	0.003	0.045	0.445	0.085	0.203	0.004	0.829	0.637	0.215
		N	330	330	330	330	330	330	330	330	330	330	330
	YiDC	R	− 0.211^a^	− 0.164^a^	0.078	− 0.048	− 0.136^b^	− 0.015	− 0.180^a^	− 0.177^a^	− 0.018	− 0.082	− 0.182^a^
		P	0.000	0.003	0.156	0.382	0.014	0.793	0.001	0.001	0.748	0.135	0.001
		N	330	330	330	330	330	330	330	330	330	330	330
	PDC	R	− 0.233^a^	− 0.240^a^	0.190^a^	0.033	− 0.135^b^	0.162^a^	− 0.371^a^	− 0.189^a^	0.247^a^	0.008	− 0.157^a^
		P	0.000	0.000	0.001	0.551	0.014	0.003	0.000	0.001	0.000	0.882	0.004
		N	330	330	330	330	330	330	330	330	330	330	330
	DHC	R	− 0.105	− 0.210^a^	0.267^a^	0.043	− 0.025	0.244^a^	− 0.230^a^	− 0.229^a^	0.144^a^	0.135^b^	− 0.058
		P	0.058	0.000	0.000	0.436	0.645	0.000	0.000	0.000	0.009	0.014	0.297
		N	330	330	330	330	330	330	330	330	330	330	330
	BSC	R	− 0.176^a^	− 0.193^a^	0.094	− 0.030	0.010	0.099	− 0.198^a^	− 0.085	0.192^a^	− 0.060	− 0.045
		P	0.001	0.000	0.089	0.584	0.855	0.072	0.000	0.123	0.000	0.279	0.418
		N	330	330	330	330	330	330	330	330	330	330	330
	QSC	R	− 0.164^a^	− 0.268^a^	0.181^a^	− 0.002	− 0.100	0.117^b^	− 0.468^a^	− 0.411^a^	0.147^a^	− 0.005	− 0.288^a^
		P	0.003	0.000	0.001	0.976	0.069	0.034	0.000	0.000	0.008	0.935	0.000
		N	330	330	330	330	330	330	330	330	330	330	330
	SDC	R	− 0.262^a^	− 0.187^a^	0.124^b^	0.093	0.021	0.103	− 0.241^a^	− 0.153^a^	0.016	− 0.012	− 0.057
		P	0.000	0.001	0.024	0.093	0.704	0.062	0.000	0.005	0.774	0.835	0.298
		N	330	330	330	330	330	330	330	330	330	330	330

Data form 319, 355 and 330 cases were used to analyze criterion validity of SF-WQ9CCMQ- Editions A, B and C, respectively

^a^Correlation is significant at the 0.01 level; ^b^Correlation is significant at the 0.05 level; R: Correliation coefficient; P: 2-tailed value; N; number of case

Abbreviations: GTC: Gentleness constitution, QDC: Qi-deficiency constitution, YaDC: Yang-deficiency constitution, YiDC: Yin-deficiency constitution, PDC: phlegm-dampness constitution, DHC: damp-heat constitution, BSC:blood-stasis constitution, QSC:Qi-stagnation constitution and ISC: inherited-special constitution; BC: body constitution; PF: Physical functioning, RP: Role limitations due to physical health, BP: Bodily pain, GH: General health perceptions, VT: Vitality, SF: Social functioning, RE: Role limitations due to emotional problems, MH: Metal health, HT: Health transition, PCS: Physical component summary, MCS: Mental component summary

##### Divergent validity

In SF-WQ9CCMQ- A, the results indicated that statistically significant differences between the obese group and the non-obese group in the PDC (P < 0.001) and DHC (P = 0.006).In SF-WQ9CCMQ- B, the results indicated that statistically significant differences between the obese group and the non-obese group in the PDC (P =0.006), QDC (P = 0.006) and YaDC (P = 0.044).And in SF-WQ9CCMQ- C, the results indicated that statistically significant differences between the obese group and the non-obese group in the PDC (P < 0.001) and YaDC (P = 0.005) (Table 8).

**Table 8:** Scores of BC in obese and the non-obese grous

Edition of short-form	BC type	BMI ≥ 25			BMI < 25			Mann–Whitney U	Wilcoxon W	Z	P-value
		Mean ± SD	Mean Rank	Sum of Ranks	Mean ± SD	Mean rank	Sum of ranks				
A	GTC	68.84 ± 15.28	163.58	46,292.00	72.32 ± 14.60	183.39	8986.00	6106.00	46,292.00	− 1.346	0.178
	QDC	24.00 ± 15.18	165.68	46,888.50	25.17 ± 16.36	171.21	8389.50	6702.50	46,888.50	− 0.378	0.705
	YaDC	26.56 ± 23.92	169.95	48,097.00	20.07 ± 19.16	146.55	7181.00	5956.00	7181.00	− 1.592	0.111
	YiDC	22.88 ± 15.58	166.33	47,072.00	22.28 ± 14.27	167.47	8206.00	6886.00	47,072.00	− 0.078	0.938
	PDC	22.20 ± 36.22	157.94	44,697.50	36.22 ± 23.67	215.93	10,580.50	4511.50	44,697.50	− 3.946	0.000**
	DHC	26.06 ± 16.37	160.54	45,432.50	34.06 ± 19.39	200.93	9845.50	5246.50	45,432.50	2.736	0.006**
	BSC	17.93 ± 15.87	167.39	47,370.00	17.86 ± 18.00	161.39	7908.00	6683.00	7908.00	− 0.411	0.681
	QSC	28.06 ± 17.68	165.49	46,835.00	29.76 ± 19.17	172.31	8443.00	6649.00	46,835.00	− 0.466	0.641
	SDC	18.93 ± 17.13	166.57	47,140.50	19.13 ± 18.37	166.00	8137.50	6912.50	8137.50	− 0.034	0.973
B	GTC	71.24± 19.84	182.92	58,717.00	75.17 ± 16.62	202.41	9918.00	7036.00	58,717.00	− 1.20	0.231
	QDC	21.26 ± 20.44	181.22	58,171.00	26.79 ± 19.93	213.55	10,464.00	6490.00	58,171.00	− 2.02	0.044
	YaDC	33.96 ± 26.83	191.38	61,434.00	22.79 ±21.64	146.96	7201.00	5976.00	7201.00	− 2.73	0.006**
	YiDC	30.61 ± 18.82	185.06	59,404.50	30.61 ± 19.79	188.38	9230.50	7723.50	59,404.50	− 0.20	0.838
	PDC	23.96 ± 19.27	174.85	56,127.50	41.33 ± 23.07	255.26	12,507.50	4446.50	56,127.50	− 4.95	0.000**
	DHC	30.56 ± 20.74	185.14	59,429.00	30.27 ± 18.38	187.88	9206.00	7748.00	59,429.00	− 0.17	0.866
	BSC	24.40 ± 19.57	186.27	59,792.50	21.94 ± 13.25	180.46	8842.50	7617.50	8842.50	− 0.36	0.720
	QSC	30.40 ± 20.14	185.52	59,552.50	29.76 ± 17.18	185.36	9082.50	7857.50	9082.50	− 0.01	0.992
	SDC	16.93 ± 19.16	182.51	58,585.50	18.19 ± 15.47	205.09	10,049.50	6904.50	58,585.50	− 1.41	0.159
C	GTC	73.63 ± 16.23	166.08	47,000.00	71.01 ± 22.13	162.02	7615.00	6487.00	7615.00	− 0.28	0.780
	QDC	23.06 ± 15.25	163.84	46,367.50	25.89 ± 18.24	175.48	8247.50	6181.50	46,367.50	− 0.79	0.431
	YaDC	27.74 ± 22.56	171.50	48,535.00	19.41 ± 22.28	129.36	6080.00	4952.00	6080.00	− 2.82	0.005**
	YiDC	26.15 ± 16.90	162.32	45,936.50	30.85± 19.35	184.65	8678.50	5750.50	45,936.50	− 1.50	0.135
	PDC	26.30 ± 19.24	153.75	43,512.00	44.86 ± 20.67	236.23	11,103.00	3326.00	43,512.00	− 5.53	0.000**
	DHC	29.04 ± 18.58	162.10	45,873.50	33.24 ± 18.98	185.99	8741.50	5687.50	45,873.50	− 1.60	0.110
	BSC	17.31 ± 16.16	165.56	46,853.00	16.84 ± 15.49	165.15	7762.00	6634.00	7762.00	− 0.03	0.978
	QSC	29.06 ± 20.06	167.11	47,291.00	25.53 ± 18.26	155.83	7324.00	6196.00	7324.00	− 0.76	0.448
	SDC	16.08 ± 17.08	162.92	46,107.00	19.50 ± 18.74	181.02	8508.00	5921.00	46,107.00	− 1.23	0.218

Data form 322, 370 and 330 cases were used to analyze divergent validity of SF-WQ9CCMQ- Editions A, B and C, respectively

Abbreviations: GTC: Gentleness constitution, QDC: Qi-deficiency constitution, YaDC: Yang-deficiency constitution,YiDC: Yin-deficiency constitution, PDC: phlegm-dampness constitution, DHC: damp-heat constitution, BSC: blood-stasis constitution, QSC: Qi-stagnation constitution and ISC: inherited-special constitution; BC: body constitution

#### Reliability evaluation

##### Test–retest reliability

The Spearman-Brown test–retest coefficient of each BC type scale is 0.638–0.851(SF-WQ9CCMQ- A), 0.684–0.854(SF-WQ9CCMQ- B) and 0.475–0.736(SF-WQ9CCMQ- C), respectively (Table 9).

**Table 9:** Reliability coefficients of 9 BC types in 3 short-forms

Edition of short-form	BC type	Item number	Cronbach’s alphacofficient	Test–retest reliability coefficient	P-value
A	Total scale	27	0.856	–	–
	GTC	4	0.665	0.841	0.000
	QDC	3	0.630	0.777	0.000
	YaDC	3	0.800	0.787	0.000
	YiDC	3	0.510	0.765	0.000
	PDC	3	0.645	0.808	0.000
	DHC	4	0.666	0.851	0.000
	BSC	3	0.530	0.638	0.000
	QSC	3	0.793	0.743	0.000
	SDC	4	0.598	0.808	0.000
B	Total scale	23	0.856	−	−
	GTC	3	0.466	0.819	0.000
	QDC	2	0.325	0.756	0.000
	YaDC	3	0.751	0.854	0.000
	YiDC	3	0.522	0.690	0.000
	PDC	3	0.499	0.831	0.000
	DHC	3	0.522	0.771	0.000
	BSC	3	0.513	0.684	0.000
	QSC	3	0.828	0.760	0.000
	SDC	3	0.544	0.818	0.000
C	Total scale	27	0.861	−	−
	GTC	2	0.464	0.496	0.000
	QDC	3	0.585	0.475	0.000
	YaDC	4	0.802	0.651	0.000
	YiDC	4	0.628	0.662	0.000
	PDC	3	0.495	0.639	0.000
	DHC	4	0.593	0.736	0.000
	BSC	3	0.389	0.722	0.000
	QSC	3	0.818	0.651	0.000
	SDC	3	0.544	0.717	0.000

Abbreviations: GTC: Gentleness constitution, QDC: Qi-deficiency constitution, YaDC: Yang-deficiency constitution, YiDC: Yin-deficiency constitution, PDC: phlegm-dampness constitution, DHC: damp-heat constitution, BSC: blood-stasis constitution, QSC: Qi-stagnation constitution and ISC: inherited-special constitution; BC: body constitution

##### Internal consistency reliability

The Cronbanch’s alpha coefficients of the total scale of 3 short-forms are 0.856, 0.856 and 0.861, respectively. Meanwhile, the Cronbanch’s alpha coefficients of each BC type scale are 0.510–0.800, 0.323–0.828, 0.389–0.818, respectively. (Table 9).

## Diagnostic validity

**Fig. 1:**
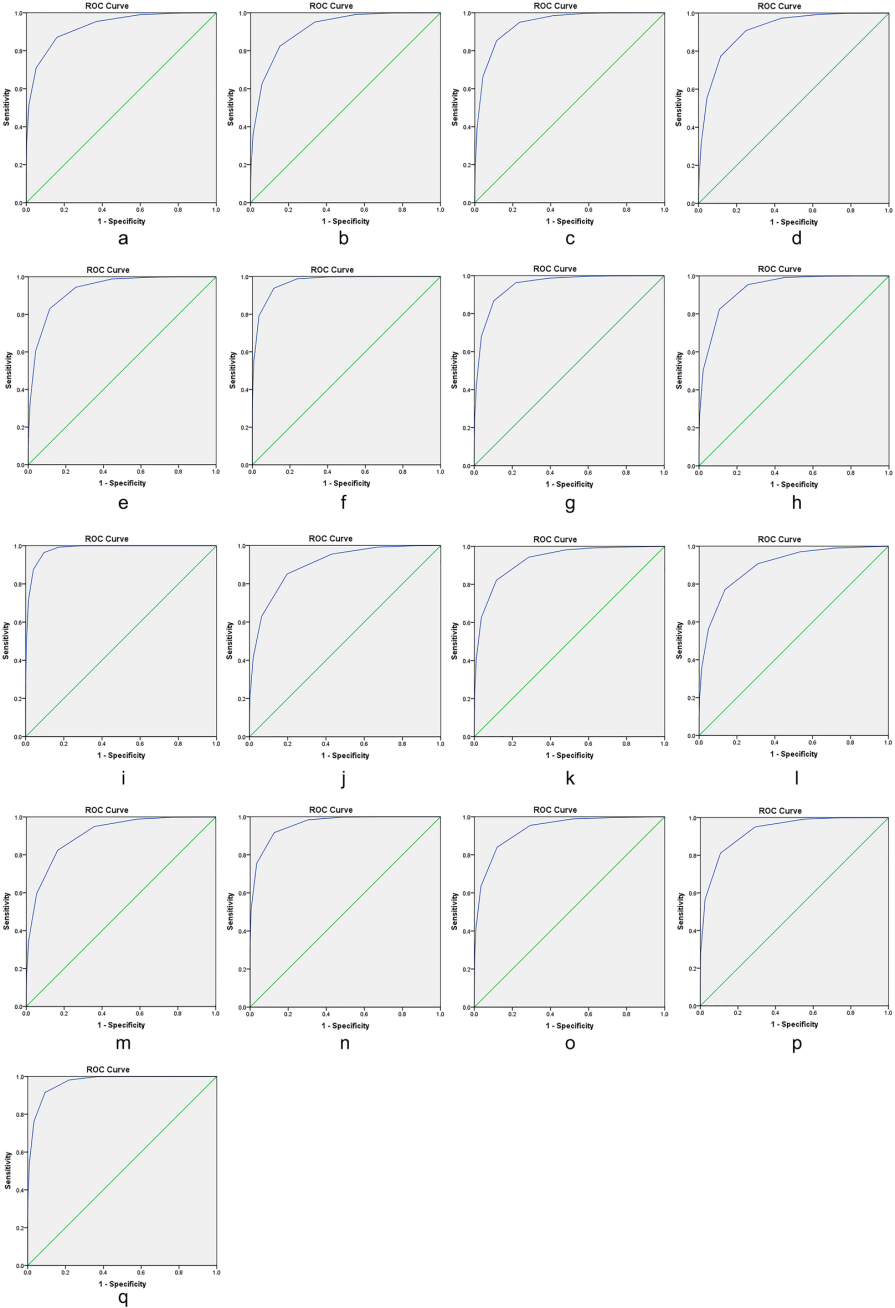
**a** Receiver operating characteristic curve for the GTC at cut-off ≥ 60. **b** Receiver operating characteristic curve for the QDC at cut-off ≥ 40. **c** Receiver operating characteristic curve for the YaDC at cut-off ≥ 40. **d** Receiver operating characteristic curve for the YiDC at cut-off ≥ 40. **e** Receiver operating characteristic curve for the PDC at cut-off ≥ 40. **f** Receiver operating characteristic curve for the DHC at cut-off ≥ 40. **g** Receiver operating characteristic curve for the BSC at cut-off ≥ 40. **h** Receiver operating characteristic curve for the QSC at cut-off ≥ 40. **i** Receiver operating characteristic curve for the SDC at cut-off ≥ 40. **j** Receiver operating characteristic curve for the QDC at cut-off ≥ 30. **k** Receiver operating characteristic curve for the YaDC at cut-off ≥ 30. **l** Receiver operating characteristic curve for the YiDC at cut-off ≥ 30. **m** Receiver operating characteristic curve for the PDC at cut-off ≥ 30. **n** Receiver operating characteristic curve for the DHC at cut-off ≥ 30. **o** Receiver operating characteristic curve for the BSC at cut-off ≥ 30. **p** Receiver operating characteristic curve for the QSC at cut-off ≥ 30. **q** Receiver operating characteristic curve for the SDC at cut-off ≥ 30

Figure 1a–q and Table 10 illustrate the detailed diagnostic validity of the best short-form—SF-WQ9CCMQ- A. The area under the ROC curve is 0.928 for the GTC scale (95%CI 0.924–0.932). Use cut-off value of the original CCMQ as 40(“yes” standard), the areas under the ROC curve are 0.895–0.969. Meanwhile, use cut-off value of the original CCMQ as 30 (“tendency” standard), the areas under the ROC curve are 0.911–0.981.

**Table 10:** The AUC value, sensitivity and specificity of each BC type

Cut-off value	BC type	AUC value	SE	P-value	95% CI		Sensitivity	Specificity
					Lower bound	Upper bound		
60	GTC	0.928	0.002	0.000	0.924	0.932	0.870	0.839
40	QDC	0.912	0.002	0.000	0.908	0.916	0.824	0.846
	YaDC	0.939	0.002	0.000	0.935	0.943	0.853	0.885
	YIDC	0.911	0.002	0.000	0.906	0.915	0.773	0.884
	PDC	0.931	0.003	0.000	0.926	0.937	0.830	0.885
	DHC	0.969	0.001	0.000	0.967	0.972	0.792	0.964
	BSC	0.949	0.002	0.000	0.945	0.953	0.867	0.898
	QSC	0.934	0.002	0.000	0.930	0.938	0.954	0.742
	SDC	0.981	0.001	0.000	0.979	0.983	0.876	0.959
30	QDC	0.900	0.002	0.000	0.896	0.905	0.851	0.803
	YaDC	0.927	0.002	0.000	0.923	0.931	0.822	0.882
	YIDC	0.895	0.002	0.000	0.890	0.899	0.770	0.864
	PDC	0.905	0.002	0.000	0.901	0.910	0.824	0.834
	DHC	0.960	0.001	0.000	0.958	0.963	0.917	0.873
	BSC	0.933	0.002	0.000	0.929	0.937	0.840	0.880
	QSC	0.929	0.002	0.000	0.926	0.932	0.951	0.709
	SDC	0.969	0.001	0.000	0.966	0.971	0.915	0.908

Abbreviations: GTC: Gentleness constitution, QDC: Qi-deficiency constitution, YaDC: Yang-deficiency constitution, YiDC: Yin-deficiency constitution, PDC: phlegm-dampness constitution, DHC: damp-heat constitution, BSC: blood-stasis constitution, QSC: Qi-stagnation constitution and ISC: inherited-special constitution; BC: body constitution; AUC: the area under the ROC curve

## Discussion

In this study, a simplified scale with qualified completion time, good validity and reliability, as well as excellent diagnostic validity was successfully developed, which through 3 steps include development of short forms, evaluation and comparison of 3 editions, diagnostic validity based on the original CCMQ. Meanwhile, there are still several issues that need further discussion.

We evaluated and compared the psychometric properties of 3 short forms comprehensively. (a) The results indicate that the SF-WQ9CCMQ- A, B, C have a similar and qualified completion time. And SF-WQ9CCMQ- A as the best version among 3 short forms, the average completion time is 63.53% (7.37 min) shorter than that of the original [22, 56]; (b) SF-WQ9CCMQ- A is the short form with the best construct validity. Although the accumulated contribution rates of all 3 short forms were > 50%, SF-WQ9CCMQ- A has the best-accumulated contribution rates of the total scale. In BC type scales of short forms, both SF-WQ9CCMQ- A and B have only one common factor which eigenvalue > 1, and accumulated contribution rates > 50% except for SDC in SF-WQ9CCMQ- A and GTC in SF-WQ9CCMQ- B. At the same time, the accumulated contributions rates of 6 BC type scales common factors in SF-WQ9CCMQ-A is higher than that in SF-WQ9CCMQ-B; (c) SF-WQ9CCMQ-A has the best criterion validity among 3 short forms. SF-WQ9CCMQ- A shows a good and regular correlation with SF36, and the criterion validity result consistent with the original CCMQ and other relevant studies [22, 61, 62], but SF-WQ9CCMQ-B and C are opposite; (d) the divergent validity results showed that there were statistically significant differences between the obese group and the non-obese group in the PDC scores of the three scales (P < 0.001). The PDC scales of 3 short-forms have good divergent validity in the BMI dimension, which is consistent with the original CCMQ and related research results [22, 61]; and (e) SF-WQ9CCMQ- Edition A shows the best test–retest reliability. Test-restest reliability > 0.7 for 8 BC type scales in SF-WQ9CCMQ- A, 7 BC type scales in SF-WQ9CCMQ- B, and 3 BC type scales in SF-WQ9CCMQ- C. Moreover, the test-restest reliability of 2 BC type scales < 0.5 in SF-WQ9CCMQ- Edition C. f)SF-WQ9CCMQ- A has the best internal consistency reliability. All the Cronbanch’s alpha coefficients were > 0.8 for the total scale of 3 short forms.But in both SF-WQ9CCMQ- B and C, there were 3 BC type scales with Cronbanch’s alpha coefficients < 0.5, respectively. At the same time, for BC type scales with Cronbanch’s alpha coefficients > 0.6, SF-WQ9CCMQ-A contains 6 BC type scales, SF-WQ9CCMQ-B contains 2 BC type scales, and SF-WQ9CCMQ-C contains 3 BC type scales. Above all, there are similar level of completion time and divergent validity among 3 short forms. Meanwhile, SF-WQ9CCMQ-A has the best Construct validity, Criterion validity, test–retest reliability and internal consistency reliability.

In the present study, considering the practical needs and high-quality concerns for the simplified version of the CCMQ, more and new attempts and method improvements were made on the basis of the previous work. (a) Adjust the participation time of expert survey. In methods 1 and 3, the expert opinions were taken account together with the results of data analysis by CTT and IRT, and after CTT and IRT selection is completed in method 2; (b) consider the weight or each item selecting method. In this study, methods 1 and 2 have the same weight of 8 indicators, and method 3 has the same weight of 3 methods (expert survey, CTT and IRT); (c) increase the discussion and review of core group and experts at the final stage. In practice, in the first method, experts put forward suggestions for item adjustment at this stage, which was finally followed in this study.

In this study, more attention and application were given to expert opinions. In the process of the development and simplification of questionnaire or scale, it was considered that expert survey should be used after data analysis [37]. However, this method has obvious disadvantages and deficiencies in practice. The items seen by experts have been screened, so they cannot see all the original items. Even if all items of the original questionnaire are provided to experts, they will also be affected by data results. Therefore, for the items with special or characteristics of TCM as well as the items that experts may consider more important or meaningful, items have been deleted before experts put forward their opinions, so they cannot be retained. In fact, expert consultation stage and final expert group discussion and approval stage of this study showed the obvious difference between expert opinions and data results.

The method is also the biggest improvement in this study such as setting the expected total number of items as a guide in the design stage and selecting only those items that meet the assumption and limit of the number of items in the selecting process. Meanwhile, all methods and indices were confirmed and applied to this study after discussion in the expert meeting at the beginning of the study. The top 3 items of each scale were retained, but the top 4 important items are selected and ranked by the experts for each BC type scale. This method ensures that the expert participants do not miss out on the comparison of the third and fourth most important items. All the above planning and improvement processes aim to ensure that these improvements can develop a short-form questionnaire with good psychometric properties and practical application value.

In the SF-WQ9CCMQ- Edition A, 1 item had been made the necessary adjustment. After discussion by the core group and the expert group, five points were made in support of this adjustment. (a) Respondents often misunderstood the meaning of item 7 in real-world practice, which might be caused by the loss of patience. Hence, they suggested deleting it; (b) the proposed increases item 1 in the original questionnaire. On the one hand, this item is the basis for judging the overall state of each people; on the other hand, an opposite direction from item 2 can be formed to verify the authenticity of filling in the questionnaire; (c) in the results of the expert survey, the item that was changed by experts obtained the highest score, which was more than twice the value of the item with the second-highest score; (d) both items 2 and 7 belong to both the QDC and GTC scales, and only one item reserved is sufficient in the GTC scale; and (e) there should be an exclusive item in the GTC scale, rather than all the other unbalance constitution of the reverse scoring items.

Modern psychometrics theory and technology are very important to promote and apply in TCM, thereby producing gratifying results—for Constitutional medicine of TCM, health management and preventive treatment of disease and many other fields provided many new instruments and results. However, obvious differences and gaps exist psychometrics measurement theory and technology with TCM such as Constitutional medicine of TCM. Whether the theory and technology of psychometric measurement could completely satisfy the development and needs of TCM should be a question worthy of attention and discussion. The systematic scientific and engineering problems exists for TCM, including the applicability of existing psychometric theories and techniques to TCM, and the way of developing more suitable psychometric measurement theories and techniques for TCM.

## Limitations

This study has some limitations. First, this study is only preliminary research. The participants' data used for psychometric properties analysis was collected from convenience sampling. Therefore, there may be some sampling error in age, gender, region, and other factors. A larger, more rigorous research should be designed and carried out. Second, the correlation and comparison research between the short-form CCMQ and other health questionnaires or scales such as SF-36/12/8, WHOQOL, WHOQOL-BEEF, and EQ5D should not be ignored in future research. Third, the original CCMQ has been widely used in health management, public health, primary care, and disease prevention and treatment. Whether the short form has the same good applicability in the above areas as the original, as well as the difference and correlation between the two instruments need to be further studied. Finally, more necessary comparative research should be carried out gradually. Such as, the original CCMQ has been translated into many languages and widely used in the world. Does the short form have the same applicability as the original? These need to be further clarified by cross-cultural research.

## Conclusions

The present study developed and evaluated a short- form CCMQ with 27 items named SF-WQ9CCMQ- A, It successfully retained the 9 BC type scale structures originally included in CCMQ. SF-WQ9CCMQ- A is the best psychometric property such as reliability and validity among 3 short forms, and has excellent diagnostic validity consistent with the original CCMQ.

This study provides a new reference and choice methods for individual and public health management questionnaire and scale development based on TCM, and offers new experience for the application and development of psychometric measurement in TCM. The results also provide an alternative instrument for rapid BC identification and large-scale screening of TCM, and a simplified questionnaire instrument for the health management of TCM in primary care.

## Data Availability

The data and materials in this study are available from the corresponding author upon request.

## Abbreviations

TCM: Traditional Chinese Medicine
CCMQ: Constitution in Chinese Medicine Questionnaire
BC: Body constitution
CACM: China Association of Chinese Medicine
GTC: Gentleness constitution
QDC: Qi-deficiency constitution
YaDC: Yang-deficiency constitution
YiDC: Yin-deficiency constitution
PDC: Phlegm-dampness constitution
DHC: Damp-heat constitution
BSC: Blood-stasis constitution
QSC: Qi-stagnation constitution
ISC: Inherited-special constitution
CTT: Classical test theory
IRT: Item response theory
CDCTCM: Classification and Determination of Constitution in TCM
EQ-CCMQ: The Expert Questionnaire of CCMQ
CVCR: Criteria value-critical ratio
SD: Standard deviation
EFA: Exploratory factor analysis
KMO: Kaiser–Meyer–Olkin
ROC: Receiver operating characteristic
AUC: The area under the ROC curve
SF-W9CCMQ: WangQi Nine body constitution questionnaire of Traditional Chinese Medicine (Short Form)
HT: Health transition
PCS: Physical component summary
MCS: Mental component summary
GH: General health perceptions
